# Supplementary material for: Effects of Thymol Supplementation on Goat Rumen Fermentation and Rumen Microbiota In Vitro
Source: Microorganisms. 2020 Jul 30;8(8):1160. doi: 10.3390/microorganisms8081160 (PMC7463607; doi:10.3390/microorganisms8081160)
Supplement: Supplementary file 1 [file microorganisms-08-01160-s001.zip › supplementary files/Supplementary File S1.docx]

**Table S1.** Effects of thymol supplementation on bacterial taxa of 7 dominant phyla and 14 dominant genera (average relative abundance > 1%) *in vitro.*

| **Phylotypes** | **Relative abundance (%)** | | | | **SEM** | ***P*-value** |
| --- | --- | --- | --- | --- | --- | --- |
|  | **CON** | **LC** | **MC** | **HC** |  |  |
| **Phyla** |  |  |  |  |  |  |
| *Bacteroidetes* | 56.00^a^ | 51.71^ab^ | 46.10^bc^ | 9.89^c^ | 4.22 | 0.006 |
| *Firmicutes* | 19.12^c^ | 23.91^bc^ | 36.46^ab^ | 71.67^a^ | 4.77 | 0.006 |
| *Synergistetes* | 7.08^ab^ | 2.65^c^ | 5.45^bc^ | 10.13^a^ | 0.71 | 0.006 |
| *Proteobacteria* | 4.05^bc^ | 10.26^a^ | 6.90^ab^ | 2.45^c^ | 1.02 | 0.011 |
| *Kiritimatiellaeota* | 5.78^a^ | 3.34^ab^ | 2.27^bc^ | 1.83^c^ | 0.39 | 0.008 |
| *Actinobacteria* | 4.49^a^ | 3.21^a^ | 1.79^b^ | 3.26^a^ | 0.27 | 0.010 |
| *Spirochaetes* | 0.67^ab^ | 3.23^a^ | 0.14^bc^ | 0.01^c^ | 0.32 | 0.006 |
| **Genera** |  |  |  |  |  |  |
| *Prevotella* 1 | 25.54^a^ | 19.06^ab^ | 10.15^bc^ | 0.87^c^ | 2.16 | 0.006 |
| *Rikenellaceae* RC9 gut group | 12.73^a^ | 10.50^ab^ | 14.52^a^ | 5.13^bc^ | 2.16 | 0.008 |
| *Succiniclasticum* | 6.28^b^ | 8.96^b^ | 9.80^b^ | 15.43^a^ | 2.16 | 0.008 |
| *Streptococcus* | 0.00^b^ | 0.00^b^ | 5.39^a^ | 30.95^a^ | 2.16 | 0.006 |
| *Pseudobutyrivibrio* | 0.10^c^ | 0.44^b^ | 5.13^ab^ | 20.49^a^ | 2.16 | 0.006 |
| *Fretibacterium* | 7.17^ab^ | 2.59^c^ | 5.46^bc^ | 10.05^a^ | 2.16 | 0.006 |
| uncultured rumen bacterium1 | 0.74^c^ | 11.23^a^ | 5.57^ab^ | 3.08^bc^ | 2.16 | 0.006 |
| *Succinivibrio* | 1.68^bc^ | 9.73^a^ | 6.19^ab^ | 1.31^c^ | 2.16 | 0.007 |
| unclassified *F082* | 4.89^a^ | 3.58^ab^ | 5.27^a^ | 0.50^b^ | 2.16 | 0.010 |
| uncultured bacterium | 3.88^ab^ | 1.70^bc^ | 7.60^a^ | 0.02^c^ | 2.16 | 0.006 |
| uncultured rumen bacterium2 | 5.71^a^ | 3.30^ab^ | 2.29^bc^ | 1.84^c^ | 2.16 | 0.007 |
| *Bifidobacterium* | 4.52^a^ | 3.19^a^ | 1.63^b^ | 2.86^a^ | 2.16 | 0.010 |
| unclassified *Lachnospiraceae* | 0.57^c^ | 1.14^bc^ | 2.81^a^ | 2.17^ab^ | 2.16 | 0.006 |
| *Veillonellaceae UCG-001* | 2.80^a^ | 2.05^ab^ | 0.88^bc^ | 0.75^c^ | 2.16 | 0.006 |

Note: SEM = standard error of the mean; CON = 0 thymol, LC = 100 mg/L thymol, MC = 200 mg/L thymol, HC= 400 mg/L thymol; In the same row, values with different superscript indicate significant differences between treatments (*P* < 0.05).

**Table S2.** Effect of thymol on alpha diversity indices of rumen bacterial, archaeal, and protozoan communities *in vitro*

| **Indices** | **CON** | **LC** | **MC** | **HC** | **SEM** | ***P*-value** |
| --- | --- | --- | --- | --- | --- | --- |
| **Bacteria** |  |  |  |  |  |  |
| Chao1 | 622.0^a^ | 524.0^ab^ | 415.0^bc^ | 218.0^c^ | 36.7 | 0.006 |
| Pielou | 0.87^a^ | 0.82^b^ | 0.80^b^ | 0.59^c^ | 0.02 | 0.006 |
| Shannon | 7.86^a^ | 7.29^b^ | 6.85^c^ | 4.54^d^ | 0.29 | 0.006 |
| **Archaea** |  |  |  |  |  |  |
| Chao1 | 95.6^ab^ | 124.0^a^ | 116.0^a^ | 52.2^b^ | 9.09 | 0.036 |
| Pielou | 0.62^b^ | 0.63^b^ | 0.66^ab^ | 0.68^a^ | 0.01 | 0.008 |
| Shannon | 4.05 | 4.30 | 4.47 | 3.86 | 0.08 | 0.081 |
| **Protozoa** |  |  |  |  |  |  |
| Chao1 | 29.8 | 36.6 | 33.2 | 19.0 | 2.18 | 0.066 |
| Pielou | 0.50^ab^ | 0.62^a^ | 0.41^b^ | 0.35^b^ | 0.03 | 0.012 |
| Shannon | 2.43^ab^ | 3.16^a^ | 2.06^b^ | 1.47^b^ | 0.17 | 0.011 |

Note: SEM = standard error of the mean; CON = 0 thymol, LC =100 mg/L thymol, MC= 200 mg/L thymol, HC= 400 mg/L thymol; In the same row, values with different superscript indicate significant differences between treatments (*P* < 0.05).

**Table S3.** Effect of thymol supplementation on rumen archaeal taxa at class and species levels *in vitro.*

| **Phylotypes** | **Relative abundance (%)** | | | | **SEM** | ***P*-value** |
| --- | --- | --- | --- | --- | --- | --- |
|  | **CON** | **LC** | **MC** | **HC** |  |  |
| **Classes** |  |  |  |  |  |  |
| *Methanobacteria* | 87.94 | 86.49 | 84.53 | 75.75 | 2.09 | 0.365 |
| unclassified *Euryarchaeota* | 8.81 | 11.12 | 13.57 | 20.81 | 1.97 | 0.260 |
| *Methanomicrobia* | 0.96 | 1.38 | 1.07 | 2.65 | 0.22 | 0.080 |
| *Thermoplasmata* | 2.21 | 0.86 | 0.75 | 0.76 | 0.24 | 0.109 |
| unclassifie*d Archaea* | 0.04 | 0.14 | 0.03 | 0.03 | 0.02 | 0.164 |
| **Species** |  |  |  |  |  |  |
| *Methanobrevibacter gottschalkii* clade | 78.54 | 75.57 | 72.69 | 68.57 | 1.87 | 0.356 |
| unclassified *Euryarchaeota* | 8.81 | 11.12 | 13.57 | 20.80 | 1.97 | 0.260 |
| *Methanobrevibacter boviskoreani* clade | 8.26^ab^ | 9.31^a^ | 9.63^a^ | 4.83^b^ | 0.57 | 0.023 |
| unclassified *Methanomicrobia* | 0.77 | 0.86 | 0.67 | 2.47 | 0.22 | 0.058 |
| *Methanosphaera sp.* Group5 | 0.21 | 0.44 | 0.56 | 1.77 | 0.20 | 0.208 |
| unclassified *Methanomassiliicoccaceae* | 1.65^a^ | 0.56^ab^ | 0.25^bc^ | 0^c^ | 0.18 | 0.008 |
| *Methanobrevibacter ruminantium* clade | 0.47^ab^ | 0.78^a^ | 1.13^a^ | 0.03^b^ | 0.13 | 0.027 |
| Group4 *sp.* MpT1 | 0.46 | 0.10 | 0.18 | 0.61 | 0.11 | 0.636 |
| *Methanosphaera sp.* ISO3-F5 | 0.34 | 0.23 | 0.30 | 0.50 | 0.07 | 0.608 |
| Group9 *sp.* ISO4-G1 | 0.08 | 0.19 | 0.28 | 0.14 | 0.07 | 0.679 |
| *Methanimicrococcus blatticola* | 0.11 | 0.28 | 0.18 | 0.04 | 0.03 | 0.095 |
| *Methanobrevibacter acididurans* | 0.10 | 0.09 | 0.23 | 0.05 | 0.04 | 0.456 |
| unclassified *Methanomicrobiales* | 0.08^a^ | 0.14^ab^ | 0.13 | 0.08^c^ | 0.02 | 0.468 |
| unclassified *Archaea* | 0.04 | 0.14 | 0.03 | 0.03 | 0.02 | 0.164 |

Note: SEM = standard error of the mean; CON = 0 thymol, LC =100 mg/L thymol, MC= 200 mg/L thymol, HC= 400 mg/L thymol; In the same row, values with different superscript indicate significant differences between treatments (*P* < 0.05).

**Table S4.** Effect of thymol supplementation on rumen protozoa at family and genus levels *in vitro.*

| **Phylotypes** | **Relative abundance (%)** | | | | **SEM** | ***P*-value** |
| --- | --- | --- | --- | --- | --- | --- |
|  | **CON** | **LC** | **MC** | **HC** |  |  |
| **Families** |  |  |  |  |  |  |
| *Ophryoscolecidae* | 87.71 | 89.35 | 94.15 | 93.60 | 1.58 | 0.365 |
| unclassified *Litostomatea* | 10.51 | 6.22 | 4.85 | 2.60 | 1.46 | 0.356 |
| *Isotrichidae* | 0.92^ab^ | 3.70^a^ | 0.65^ab^ | 0^b^ | 0.46 | 0.071 |
| unclassified *Entodiniomorphida* | 0.86 | 0.73 | 0.34 | 0.56 | 0.11 | 0.365 |
| **Genera** |  |  |  |  |  |  |
| *Entodinium* | 85.08 | 80.33 | 91.73 | 90.48 | 1.77 | 0.103 |
| unclassified *Litostomatea* | 10.51 | 6.22 | 4.85 | 2.60 | 1.46 | 0.356 |
| *Enoploplastron* | 0.40 | 2.49 | 2.03 | 2.92 | 0.39 | 0.110 |
| *Isotricha* | 0.85 | 3.55 | 0.40 | 0 | 0.43 | 0.060 |
| *Diploplastron-Eremoplastron* | 1.21^ab^ | 3.37^a^ | 0.01^b^ | 0.10^b^ | 0.40 | 0.016 |
| unclassified *Entodiniomorphida* | 0.86 | 0.73 | 0.34 | 0.56 | 0.11 | 0.365 |
| *Polyplastron* | 0.42^ab^ | 1.97^a^ | 0.08^ab^ | 0^b^ | 0.32 | 0.029 |
| *Epidinium* | 0.03 | 0.75 | 0 | 0 | 0.12 | 0.077 |
| *Ophryoscolex* | 0.30 | 0.37 | 0 | 0.09 | 0.07 | 0.239 |
| unclassified *Ophryoscolecidae* | 0.27 | 0.06 | 0.29 | 0 | 0.08 | 0.260 |

Note: SEM = standard error of the mean; CON = 0 thymol, LC =100 mg/L thymol, MC= 200 mg/L thymol, HC= 400 mg/L thymol; In the same row, values with different superscript indicate significant differences between treatments (*P* < 0.05).


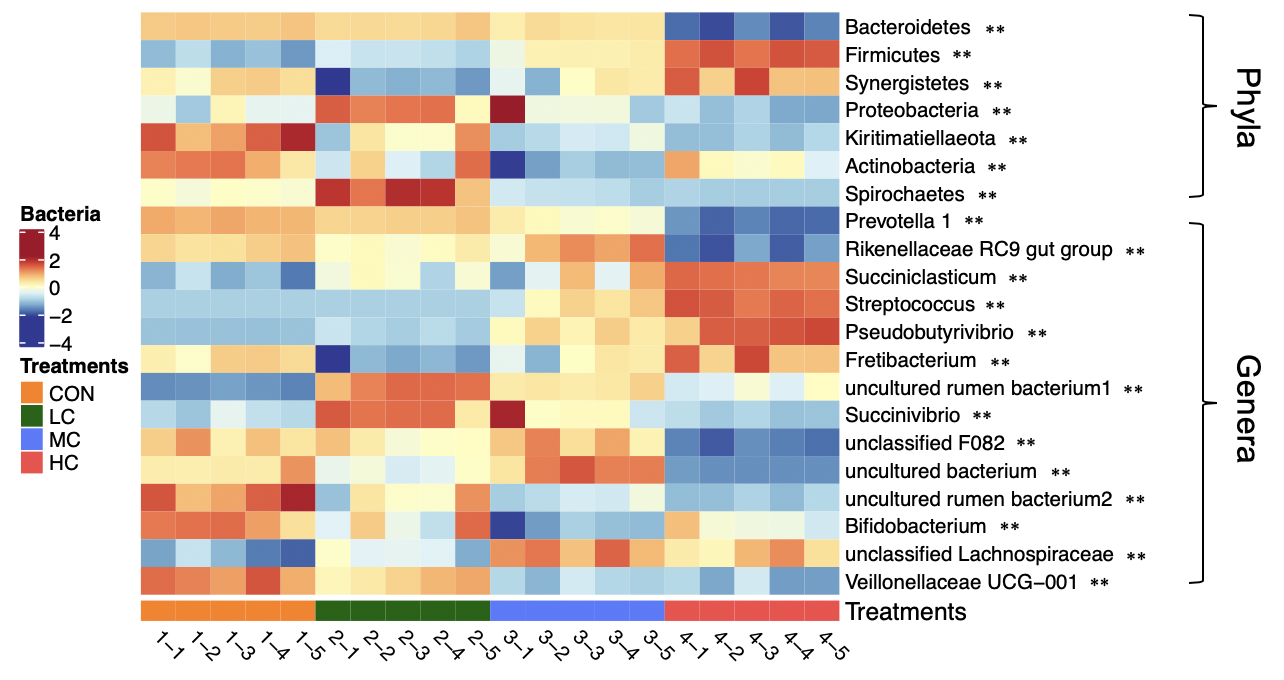


**Figure S1.** Differentially abundant rumen bacterial taxa at phylum and genus levels with different doses of thymol supplementation in heatmap. The relative abundances were log10 transformed and then normalized to generate the final matrix for heatmap plot. Significant effects of thymol supplementation were marked with ns, *P* ≥ 0.05; *, *P* < 0.05; **, *P* < 0.01; and ***, *P* < 0.001 (Kruskal-Wallis test, *P*-values were adjusted with BH method)


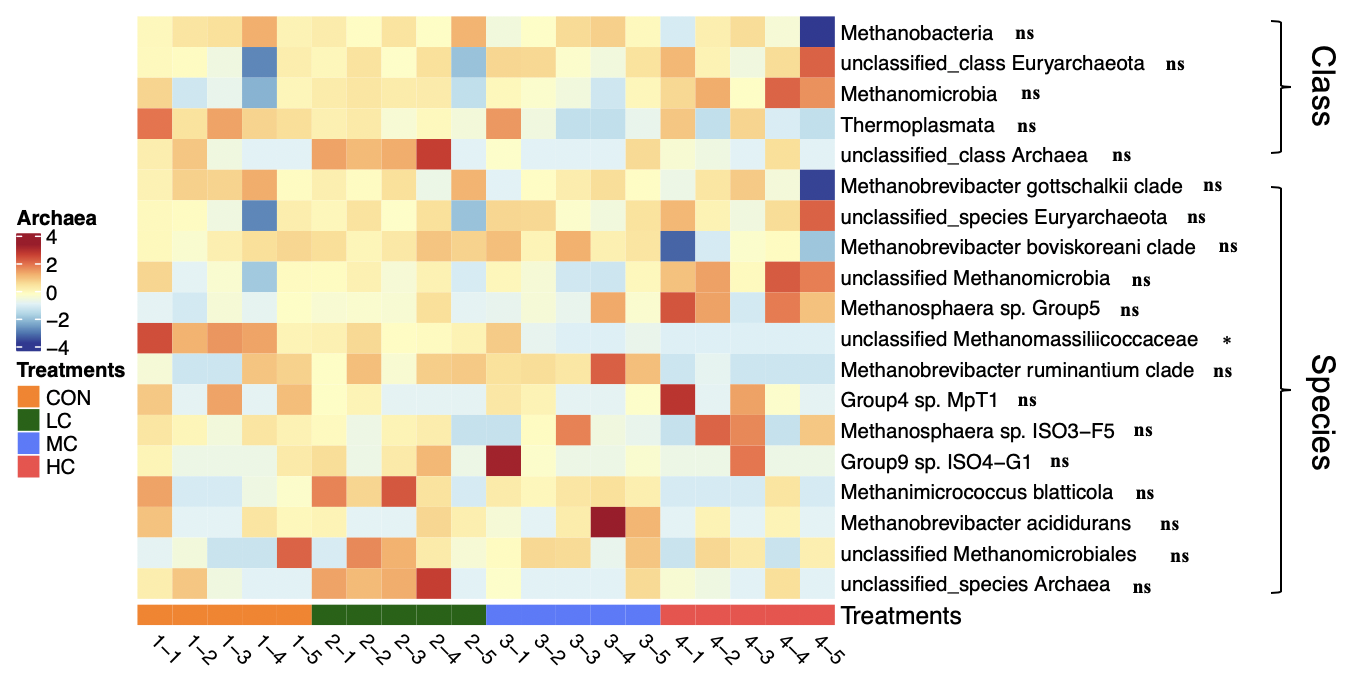


**Figure S2.** Differentially abundant rumen archaeal taxa at class and species levels with different doses of thymol supplementation in heatmap. The relative abundances were log10 transformed and then normalized to generate the final matrix for heatmap plot. Significant effects of thymol supplementation were marked with ns, *P* ≥ 0.05; *, *P* < 0.05; **, *P* < 0.01; and ***, *P* < 0.001 (Kruskal-Wallis test, *P*-values were adjusted with BH method)
