# Supplementary material for: Effects of Thymol Supplementation on Goat Rumen Fermentation and Rumen Microbiota In Vitro
Source: Microorganisms. 2020 Jul 30;8(8):1160. doi: 10.3390/microorganisms8081160 (PMC7463607; doi:10.3390/microorganisms8081160)
Supplement: Supplementary file 1 [file microorganisms-08-01160-s001.zip › supplementary files/Classifiers_trainning in QIIME2.rtf]

Train a classifier using naive-bayes methodFor archaea(RIM-DB classifier training)qiime tools import --type 'FeatureData[Sequence]' --input-path RIM_DB.fasta --output-path RIM_DB.qzaqiime feature-classifier extract-reads --i-sequences RIM_DB.qza --p-f-primer AGGAATTGGCGGGGGAGCAC --p-r-primer GCGGTGTGTGCAAGGAGC --p-min-length 400 --p-max-length 600 --o-reads ref-RIM_DB.qzaqiime tools import --type 'FeatureData[Taxonomy]' --input-format HeaderlessTSVTaxonomyFormat --input-path RIM_DB_14_07_c_revised.txt --output-path RIM_DB_14_07_c_revised.qzaqiime feature-classifier fit-classifier-naive-bayes --i-reference-reads ref-RIM_DB.qza --i-reference-taxonomy RIM_DB_14_07_c_revised.qza --o-classifier RIM_DB_14_07_c_revised_classifier.qzaFor ciliate protozoa classifier trainingqiime tools import --type 'FeatureData[Sequence]' --input-path ciliate_protoza.fasta --output-path ciliate_protozoa.qzaqiime feature-classifier extract-reads --i-sequences ciliate_protozoa.qza --p-f-primer GACTAGGGATTGGAGTGG --p-r-primer AATTGCAAAGATCTATCCC --p-min-length 300 --p-max-length 600 --o-reads ref-ciliate_protoza.qzaqiime tools import --type 'FeatureData[Taxonomy]' --input-format HeaderlessTSVTaxonomyFormat --input-path ciliates_protozoa_taxonomy.txt --output-path ciliates_protozoa_taxonomy.qzaqiime feature-classifier fit-classifier-naive-bayes --i-reference-reads ref-ciliate_protoza.qza --i-reference-taxonomy ciliates_protozoa_taxonomy.qza --o-classifier ciliates_protozoa_classifier.qza
